# Recombinant Turkey Herpesvirus Expressing H9N2 HA Gene at the HVT005/006 Site Induces Better Protection Than That at the HVT029/031 Site

**DOI:** 10.3390/v14112495

**Published:** 2022-11-11

**Authors:** Xusheng Zai, Bin Shi, Hongxia Shao, Kun Qian, Jianqiang Ye, Yongxiu Yao, Venugopal Nair, Aijian Qin

**Affiliations:** 1 Ministry of Education Key Laboratory for Avian Preventive Medicine, Yangzhou University, No.12 East Wenhui Road, Yangzhou 225009, China; 2 Jiangsu Co-Innovation Center for Prevention and Control of Important Animal Infectious Diseases and Zoonoses, No.12 East Wenhui Road, Yangzhou 225009, China; 3 Joint International Research Laboratory of Agriculture and Agri-Product Safety, The Ministry of Education of China, Yangzhou 225009, China; 4 The Pirbright Institute & UK-China Centre of Excellence for Research on Avian Diseases, Pirbright, Ash Road, Guildford, Surrey GU24 0NF, UK

**Keywords:** HVT vectored vaccine, HVT005/006, HVT029/031, H9N2, hemagglutinin

## Abstract

Turkey herpesvirus (HVT) is widely used as an effective recombinant vaccine vector for expressing protective antigens of multiple avian pathogens from different loci of the HVT genome. These include the HVT029/031 (UL22–23) locus for the insertion of IBDV VP2 and the recently identified HVT005/006 locus as a novel site for expressing heterologous proteins. In order to compare the efficacy of recombinant vaccines with the HA gene at different sites, the growth curves and the HA expression levels of HVT-005/006-hCMV-HA, HVT-005/006-MLV-HA, and HVT-029/031-MLV-HA were first examined in vitro. While the growth kinetics of three recombinant viruses were not significantly different from those of parent HVT, higher expression of the HA gene was achieved from the HVT005/006 site than that from the HVT029/031 site. The efficacy of the three recombinant viruses against avian influenza H9N2 virus was also evaluated using one-day-old SPF chickens. Chickens immunized with HVT-005/006-MLV-HA or HVT-005/006-hCMV-HA displayed reduced virus shedding compared to HVT-029/031-MLV-HA vaccinated chickens. Moreover, the overall hemagglutination inhibition (HI) antibody titers of HVT-005/006-HA-vaccinated chickens were higher than that of HVT-029/031-HA-vaccinated chickens. However, HVT-005/006-MLV-HA and HVT-005/006-hCMV-HA did not result in a significant difference in the level of HA expression in vitro and provided the same protective efficacy (100%) at 5 days after challenge. In the current study, the results suggested that recombinant HVT005/006 vaccines caused better expression of HA than recombinant HVT029/031 vaccine, and that HVT-005/006-MLV-HA or HVT-005/006-hCMV-HA could be a candidate vaccine for the protection of chickens against H9N2 influenza.

## 1. Introduction

Turkey herpesvirus (HVT) is a widely used recombinant vaccine vector for expressing heterologous antigenic proteins for the control of several avian diseases [1,2,3,4] by providing longstanding immunity in vaccinated chickens [5]. HVT has been shown to be very good at inducing immune responses, even in the presence of maternal antibodies, which is a major advantage for mass vaccination strategies compared with inactivated vaccines and other live viral vectored vaccines such as fowl pox virus (FPV)-based vaccines [6] and Newcastle disease virus (NDV)-based vaccines [7]. The HVT genome, which is about 159 kb of double-stranded DNA, contains several non-essential regions for viral replication. Some of these non-essential genes, such as US2 and US10, have been used for the generation of recombinant viruses by replacing a part of the sequence with the foreign antigen gene expression cassette. The US2 site has been used for the insertion of the HA (AIV), VP2 (IBDV), and F (NDV) genes while the US10 site has been reported for the HA (AIV) or IL-2 gene insertion [8,9,10,11]. Except for the non-essential genes of the HVT genome, the intergenic regions such as UL45/UL46 and HVT065/HVT066 were also considered as sites for the insertion of foreign genes [11]. For the insertion sites involved in this study, the HVT005/006 site was reported as a novel potential site for expressing heterologous protein [12], while the HVT029/031 (UL22–23) site was used for generating recombinant HVT-VP2 [13].

Avian influenza viruses (AIV) have become globally widespread in poultry and have caused substantial economic loss [14]. H9N2 avian influenza viruses, as the low-pathogenicity avian influenza viruses (LPAIV), receive less attention compared with H5 and H7 AIVs. However, H9N2 has become the dominant AIV subtype in chickens in China and poses an increased risk for human infections as almost all of the H9 AIVs prefer human-type receptors [15]. Most cases of human fatal infection by H7N9 have a history of contact with live poultry [16]. The coexistence of H7N9 and H9N2 in chickens could potentially generate a more virulent strain that infects humans at any time through reassortment due to the segmented nature of influenza viruses [17,18]. In order to prevent and control H9N2 AIVs, the Chinese government implemented a long-term vaccination program (inactivated vaccines) in chicken farms as early as 1998 [7]. Although various approaches were taken to control H9N2 AIVs, the outbreaks caused by H9N2 viruses in poultry are still not efficiently controlled [8]. To solve this problem, there is a need to develop new vaccines against H9N2 to protect domestic poultry in China; currently, HVT vector vaccines are the tool of choice.

In this study, we compared the efficacy of the recombinant vaccines expressing the HA gene at two sites. We examined the growth kinetics and HA expression levels of HVT-005/006-hCMV-HA, HVT-005/006-MLV-HA, and HVT-029/031-MLV-HA in vitro and evaluated the immune protection efficiency of three recombinant viruses. The effects of different promoters, the human cytomegalovirus (hCMV) promoter and the murine leukemia virus (MLV) promoter, were also evaluated for driving HA gene expression. Moreover, we also evaluated the protection of these three recombinant viruses against H9 AIV challenge. Our results showed that the HVT005/006 site was better than the HVT029/031 site for HVT-HA generation and the MLV promoter and the hCMV promoter were not significantly different in driving HA gene expression in the recombinant viruses.

## 2. Materials and Methods

### 2.1. Viruses and Cell Culture

A/Chicken/China/H1/2019 (H9N2) [19] was isolated and stored at Key Laboratory of Jiangsu Preventive Veterinary Medicine. Primary chicken embryo fibroblasts (CEFs) were prepared from 9-day-old embryos. CEFs were cultured in M199 medium (Thermo Fisher Scientific, Shanghai, China) with 5% fetal bovine serum (Thermo Fisher Scientific, Shanghai, China), 10% tryptose phosphate broth (Thermo Fisher Scientific, Shanghai, China), and 100 units/mL of penicillin and streptomycin (Thermo Fisher Scientific, Shanghai, China).

### 2.2. Generation of the Recombinant HVT-HA Viruses

HVT-005/006-MLV-HA was generated previously [12]. The process of generating recombinant HVT-029/031-MLV-HA and HVT-005/006-hCMV-HA was the same as previously described [12]. Briefly, after transfection and infection, the GFP marker was used for plaque purification to obtain HVT-HA-GFP. HVT-HA was generated by the excision of GFP using Cre recombinase.

### 2.3. Viral Growth Kinetics

To compare the growth properties of HVT-005/006-MLV-HA, HVT-029/031-MLV-HA, and HVT-005/006-hCMV-HA, CEFs in 6-well plates were infected with 100 PFU per well of HVT or the recombinant HVT viruses. CEFs were harvested at 24, 48, 72, 96, and 120 h post infection (hpi) for DNA extraction using the DNeasy 96 Blood and Tissue Kit (Qiagen, Shanghai, China). The viral copies were determined by real-time qPCR analysis to generate the growth curves of the viruses, as described previously [20,21]. Briefly, a duplex real-time qPCR was carried out to detect the HVT SORF1 gene and the chicken ovotransferrin (OVO) gene to calculate the number of HVT genome copies per 10,000 cells. Ten-fold dilution series of pGEM-T-SORF1 and pGEM-T-OVO was used to generate standard curves, respectively. The number of HVT genome copies per 10,000 cells was plotted against hours post-infection for each of the viruses.

### 2.4. HA Expression of the Recombinant HVT Viruses In Vitro

To compare the HA expression of HVT-005/006-MLV-HA, HVT-029/031-MLV-HA, and HVT-005/006-hCMV-HA in vitro, CEFs in 6-well plates were infected with HVT or the recombinant HVT viruses. CEFs were harvested at 72 h post infection and the relative expression levels of the HA and SORF1 were determined by RT-qPCR (7500 Real-Time PCR System, 7500 Software v2.3, Thermo Fisher Scientific, Carlsbad, CA, USA) as reported previously [22]. The total RNA from cells infected with viruses was prepared by the AxyPrep™ Multisource Total RNA Miniprep kit (AXYGEN, Hangzhou, China) and 1 μg RNA was used in reverse-transcription reaction by PrimeScript RT Master Mix (TaKaRa, Dalian, China) following the manufacturer’s instructions. Then, 1 μL diluted cDNA, 400 nM primers, and 10 μL SYBR Green Master Mix were used for the real-time PCR in a final volume of 20 μL. The amplification conditions were as follows: 95 °C for 30 s, followed by 40 cycles of 95 °C for 5 s and 60 °C for 34 s. The gene expression levels were normalized to the level of HVT SORF1 mRNA. The analysis of the relative gene expression data was performed by using the 2−ΔΔCT method. Meanwhile, CEFs infected with HVT or the recombinant HVT viruses were also harvested at 72 h post-infection and the relative expression levels of the HA and gB protein were determined by Western blot as reported previously [12]. Non-reducing SDS-PAGE was used to analyze the 100–130 kD gB and 75 kD HA0. The protein expression levels were normalized to the level of HVT gB protein. The grayscale analysis of the relative protein expression data was performed by using ImageJ (version 1.51j8) software 

### 2.5. Immunization and Bird Challenge Experiments

To examine the protective efficacy of HVT-005/006-MLV-HA, HVT-005/006-hCMV-HA, and HVT-029/031-MLV-HA, one-day-old SPF chickens (Boehringer Ingelheim Vital Biotechnology Co., Ltd., Beijing, China) were randomly divided into 5 groups; each group included 10 chickens. Group 1, group 2, and group 3 was vaccinated with 5000 PFU each of HVT-005/006-MLV-HA, HVT-005/006-hCMV-HA, or HVT-029/031-MLV-HA per dose, respectively. The chickens in group 4 and group 5 were inoculated with parental HVT. Randomly, blood samples were collected weekly from 5 chickens per each group until 28 days post vaccination (dpv). Serum antibody titers specific for H9N2 A/Chicken/China/H1/2019 were measured by the hemagglutinin inhibition (HI) assay. Chickens were challenged with A/Chicken/China/H1/2019 (H9N2) at 0.1 mL of 10^8^ EID_50_ at 28 days post vaccination (0 days post challenge, dpc). Oropharyngeal and cloacal swabs were collected at 3 and 5 dpc. The viral detection of swab samples was performed by inoculation of the mixture of oropharyngeal and cloacal swabs from each chicken into 2 chicken embryos. Allantoic fluid samples were analyzed by hemagglutination assay. The design of the animal experiment is shown in Table 1.

### 2.6. Hemagglutination Inhibition Assay

Hemagglutination inhibition (HI) assay was performed to determine the immunogenicity of recombinant HVT viruses as described previously [23]. Briefly, two-fold dilutions of chicken serum samples were tested in duplicate in 96-well V-bottomed plates, followed by adding 4 hemagglutination units (HAU) of H9N2 challenge virus diluted in PBS. After plates were incubated at room temperature for 0.5 h, 0.5% chicken red blood cells was added to the virus/serum mixture and incubated at room temperature for another 30 min. The hemagglutination inhibition (HI) antibody titer was determined as the reciprocal of the highest dilution that completely prevented RBC from agglutination.

### 2.7. Statistical Analyses

Differences between different groups were compared and analyzed using Prism software (GraphPad) by unpaired *t*-test and the difference was considered statistically significant at *p* < 0.05.

## 3. Results

### 3.1. Generation of the Recombinant HVT-HA Viruses

**Figure 1:**
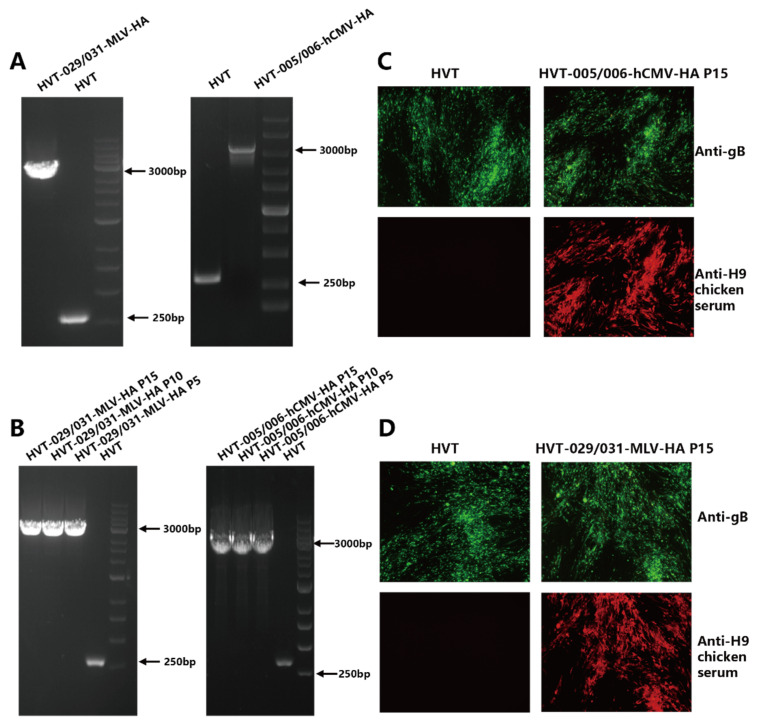
Characterization of the recombinant HVT-029/031-MLV-HA and HVT-005/006-hCMV-HA. (**A**) PCR analysis of the inserted HA gene cassette in the HVT005/006 or HVT029/031 site. (**B**) PCR analysis to confirm the presence of HA expression cassette from HVT-029/031-MLV-HA and HVT-005/006-hCMV-HA at passage 5, 10, and 15 in CEFs using primer pairs outside of the insertion site. (**C**) Detection of HA expression from HVT-005/006-hCMV-HA at passage 15 in CEFs by IFA using anti-H9 chicken serum. HVT infection was confirmed by IFA with anti-gB monoclonal antibody BD8. (**D**) Detection of HA expression from HVT-029/031-MLV-HA at passage 15 in CEFs by IFA using anti-H9 chicken serum. HVT infection was confirmed by IFA with anti-gB monoclonal antibody BD8.

HVT-005/006-MLV-HA was generated previously and the recombinant HVT-029/031-MLV-HA and HVT-005/006-hCMV-HA were generated in the same way as described in our previous article. Briefly, the HA expression cassette, along with a fluorescent marker GFP, was inserted into the HVT genome to generate HVT-HA-GFP. Then, the GFP was removed by the Cre recombinase to obtain recombinant HVT-HA. The identification of HVT-029/031-MLV-HA and HVT-005/006-hCMV-HA is shown in Figure 1. Correct insertion was examined by PCR with primers located outside of the insertion site. In Figure 1A, only the HA cassette band (about 3500 bp) was obtained in HVT-029/031-MLV-HA and HVT-005/006-hCMV-HA, with no DNA band for the wild-type virus. Recombinant HVT-HA viruses were passaged in CEF cells for 15 passages and genetic stability of the HA expression cassette was detected by PCR using viral DNA extracted from every 5 passages. The expression of the HA gene was determined by IFA at the 15th passage. PCR results (Figure 1B) showed that only the HA cassette was amplified from DNA samples taken from passages 5, 10, and 15 of the recombinant HVT-HA viruses whereas no band was observed from wild-type HVT. The HA protein expression was detected by indirect immunofluorescence analysis (IFA). In Figure 1C,D, HA protein expression can be observed in recombinant virus-infected cells in contrast to cells infected with wild-type HVT. However, the HVT-gB was detected in both HVT and recombinant HVT virus-infected cells.

### 3.2. Growth Kinetics of Different Recombinant HVT-HA Viruses In Vitro

**Figure 2:**
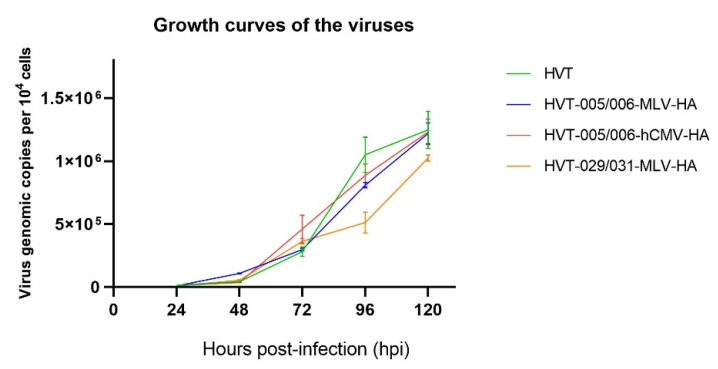
Growth curves of recombinant HVT viruses and HVT. Viral genomic copies per 10^4^ cells, based on the HVT SORF1 gene, were determined by real-time qPCR on DNA from virus-infected CEFs sampled at 24, 48, 72, 96, and 120 h post infection. Differences between data for the parental HVT and the recombinant HVT viruses were analyzed by unpaired *t*-test and no significant difference was found (*p* > 0.05).

The growth kinetics of HVT-005/006-MLV-HA, HVT-029/031-MLV-HA, and HVT-005/006-hCMV-HA were measured to determine whether there were any differences in the in vitro replication of the recombinant virus compared with HVT. Statistical analysis of the differences between the parental HVT and the recombinant HVT viruses was analyzed by an unpaired *t*-tests and no significance was found (*p* > 0.05). Meanwhile, as demonstrated in Figure 2, the growth curves of HVT-HA viruses were not significantly different from that of wild-type HVT.

### 3.3. Comparison of HA Expression of Different Recombinant HVT-HA Viruses In Vitro

**Figure 3:**
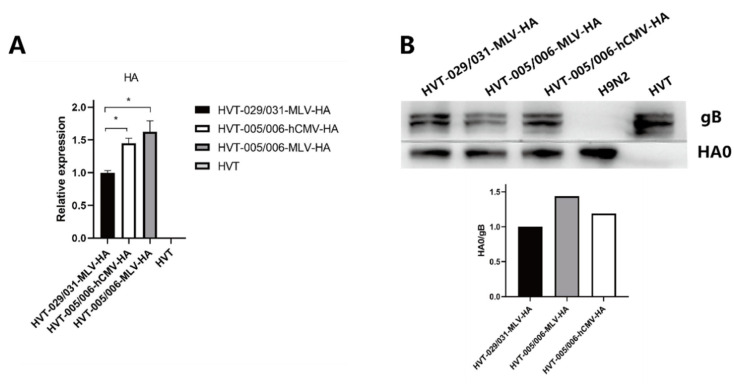
(**A**) Relative expression levels of HA mRNAs in CEF cells infected with HVT or recombinant HVT viruses. Asterisks (*) indicate a statistically significant difference. *, *p* < 0.05. (**B**) Detection of HA protein expression by western blot and grayscale analysis by western blot.

To investigate the HA expression of HVT-005/006-MLV-HA, HVT-029/031-MLV-HA, and HVT-005/006-hCMV-HA in vitro, CEFs infected with HVT or the recombinant HVT viruses were harvested at 72 h post infection. The relative expression levels of HA and SORF1 were determined by real-time PCR. The HA gene expression levels were not significantly different between HVT-005/006-MLV-HA and HVT-005/006-hCMV-HA, but some higher compared to that of HVT-029/031-MLV-HA (Figure 3A). Meanwhile, the experssion of HA protein and gB protein was determined by western blotting (Figure 3B). The results of the grayscale analysis showed that the HA protein expression level of HVT-005/006-MLV-HA was 1.4 times higher than that of HVT-029/031-MLV-HA.

### 3.4. Protective Efficacy of Recombinant HVT-HA Viruses against AIV H9N2 in Chickens

To determine the protective efficacy of recombinant HVT-005/006-MLV-HA, HVT-029/031-MLV-HA, and HVT-005/006-hCMV-HA against challenge with H9N2, one-day-old chickens were immunized with the three recombinant viruses. Chickens were challenged with 10^8^ EID_50_ of A/Chicken/China/H1/2019 (H9N2) by intravenous injection at 28 day after vaccination. Protection was determined in terms of virus shedding. At 3 days post challenge, 8/10 of chickens vaccinated with HVT-005/006-MLV-HA were protected while HVT-005/006-hCMV-HA provided 9/10 protective efficacy. Compared with HVT-005/006-MLV-HA and HVT-005/006-hCMV-HA, the protective efficacy was only 7/10 for HVT-029/031-MLV-HA. At 5 days post challenge, all chickens vaccinated with HVT-005/006-MLV-HA or HVT-005/006-hCMV-HA were protected, while 7/9 of chickens vaccinated with HVT-029/031-MLV-HA were protected (Table 2).

### 3.5. Antibody Responses against H9N2 Virus Induced by Recombinant HVT-HA Viruses in Vaccinated Chickens

HA antibodies play an important role in the defense against viral infections; therefore, the HI assay of vaccinated chicken serum was carried out to examine the capacity of three recombinant viruses to induce a protective humoral immune response (Table 3). The results showed that the earliest immune responses to the HA protein were detected at 14 days post vaccination and the Log_2_ HI titers of the three recombinant viruses were not significantly different at 14 days post vaccination. HI titers of the sera from HVT-005/006-MLV-HA and HVT-005/006-hCMV-HA-vaccinated chicken were higher than that of HVT-029/031-MLV-HA-vaccinated chicken at 21 days post vaccination and 28 days post vaccination. The highest Log_2_ HI titer (4.8 ± 0.84) was observed in HVT-005/006-MLV-HA vaccinated chickens at 28 days post vaccination. Group 4 showed no evidence of HI antibody responses.

## 4. Discussion

In this study, three recombinant HVT viruses (HVT-005/006-hCMV-HA, HVT-005/006-MLV-HA, and HVT-029/031-MLV-HA) were used to infect CEF cells to compare their characteristic in vitro. The results of the in vitro viral growth curve demonstrated that the growth of three recombinant HVT viruses was not significantly different to parental HVT, indicating that HA insertion in the HVT005/006 site or HVT029/031 site did not affect the replication of the recombinant viruses (the difference between the data for HVT and the recombinant HVT viruses was not statistically significant (*p* > 0.05)). HA expression levels of three recombinant HVT viruses in vitro were determined by quantitative RT-PCR; HVT-005/006-hCMV-HA or HVT-005/006-MLV-HA had a higher level of HA expression than HVT-029/031-MLV-HA, about a 1.5-fold increase. However, the expression level of the HA gene driven by the MLV promoter and the hCMV promoter was not significantly different (data did not show a significant difference (*p* > 0.05)). In addition, the HA protein expression level of HVT-005/006-MLV-HA was 1.4 times higher than that of HVT-029/031-MLV-HA. These results demonstrated that the HVT005/006 site was better for HA gene expression than the HVT029/031 locus, while the MLV promoter had little difference to the hCMV promoter.

Differences between the insertion sites in relation to the levels of antigen expression could affect the protective efficacy of the vaccine, as confirmed by many researchers. For the different insertion sites, Gao et al. compared the effects of inserted genes in the US2 and US10 sites on the efficacy of the recombinant vaccines. rHVT-US2-HA-vaccinated chickens were better protected than rHVT-US10-HA-vaccinated chickens following the HPAIV challenge [9]. For the different promoters, Tsukamoto et al. constructed two recombinant HVT viruses expressing VP2 (IBDV) with the cytomegalovirus (CMV) promoter and the CMV/β-actin chimera (Pec) promoter. They found that rHVT-Pec-VP2 expressed VP2 approximately four-fold more than rHVT-CMV-VP2 in vitro and induced complete protection, whereas rHVT-CMV-VP2 induced 58% protection [3]. Li et al. also compared the CMV promoter and the Pec promoter. In their study, recombinant rMDV1-VP2 with the Pec promoter expressed a larger amount of VP2 and provided complete protection against vvIBDV challenge in chickens compared with rMDV1-VP2 with the CMV promoter [24]. Similarly, our results also confirmed this. HVT-005/006-MLV-HA and HVT-005/006-hCMV-HA, with higher HA gene expression in vitro compared with HVT-029/031-MLV-HA, provided better protective efficacy: 8 out of 10 chickens vaccinated with HVT-005/006-MLV-HA and 9 out of 10 chickens vaccinated with HVT-005/006-hCMV-HA were protected at 3 days post challenge and all the chickens vaccinated with these two recombinant viruses were protected at 5 days post challenge. On the contrary, HVT-029/031-MLV-HA provided lower protective efficacy (7/10 at 3 days post challenge and 7/9 at 5 days post challenge).

HA antibodies had a significant effect on the defenses against viral infections and the specific hemagglutination inhibition titer was used to assess the humoral immune responses to AI [25]. In rDEV-∆UL2-HA-vaccinated ducks with good vaccine efficacy, Log_2_ HI antibody titers were higher than 8 at 4 weeks post vaccination while some ducks with titers ranging from 3 to 4 were only partially protected against the H9N2 AIV virus [26]. Research by Gao et al. and Li et al. also confirmed this: 26.7% of chickens vaccinated with rHVT US10-HA were protected (Log_2_ HI antibody titers at 28 days post infection were 3) whereas 60% of the chickens vaccinated with rHVT-US2-HA were protected (Log_2_ HI antibody titers were 4) [9]. Li et al. performed the vaccine efficacy experiment for rHVT-H7HA and found the two vaccinated birds that died on day 6 post challenge had relatively low to moderate levels of HI and VN titers when compared with the five survivors [2]. In our study, Log_2_ HI antibody titers in vaccinated chickens ranged from 4.2 ± 1.1 to 4.8 ± 0.84 at 28 days post infection and HVT-005/006-MLV-HA or HVT-005/006-hCMV-HA induced higher humoral immune responses than HVT-029/031-MLV-HA due to the higher Log_2_ HI antibody titers. It is worth noting that chickens vaccinated with recombinant HVT viruses provided good protection even the Log_2_ HI antibody titers were not high. This could be attributed to cellular immunity which is another important factor involved in the protection of chickens confirmed by other researchers. rHVT-H9 could induce robust cell immune responses in vaccinated chickens compared with HVT [27] and rHVT-H5 vaccine could also generate both humoral and cell-mediated immune responses [28,29]. The recombinant viruses in this study, especially HVT-005/006-MLV-HA and HVT-005/006-hCMV-HA, providing 100% protective efficacy at 5 days post challenge, could be a candidate vaccine for protection against H9N2 influenza.

In summary, we compared the effects of different loci in expressing the HA gene on the efficacy of the recombinant vaccines. HVT-005/006-MLV-HA and HVT-005/006-hCMV-HA led to better HA expression in vitro and provided better immune protection in chickens than HVT-029/031-MLV-HA. Our results showed that the HVT005/006 site was better than the HVT029/031 site when generating recombinant HVT expressing HA protein, meanwhile, HVT-005/006-MLV-HA or HVT-005/006-hCMV-HA, providing good protective efficacy, could be candidate vaccines against H9N2 influenza.

## Figures and Tables

**Table 1:** Outline of the animal experiment.

Group*	Vaccination (1-Day-Old Chickens)	Vaccine Dose (PFU)	Challenge Infection (28-Day-Old Chickens)	Challenge Dose (EID_50_)
1	HVT-005/006-MLV-HA	5000	A/Chicken/China/H1/2019(H9N2)	10^8^
2	HVT-005/006-hCMV-HA	5000	A/Chicken/China/H1/2019(H9N2)	10^8^
3	HVT-029/031-MLV-HA	5000	A/Chicken/China/H1/2019(H9N2)	10^8^
4	HVT	5000	A/Chicken/China/H1/2019(H9N2)	10^8^
5	HVT	5000	/	/

Asterisk (*): Each group included 10 chickens.

**Table 2:** Virus detection in swabs of vaccinated and mock-vaccinated chickens challenged with H9N2 at different time points.

Vaccination	Virus Isolation from Swabs (Shedding/Total)	
	3 (Days Post Challenge, dpc)	5 (dpc)
HVT-005/006-MLV-HA *	2/10	0/10
HVT-005/006-hCMV-HA *	1/10	0/10
HVT-029/031-MLV-HA *	3/10	2/10
HVT *	10/10	9/10
HVT	0/10	0/10

Asterisks (*) represent that the immunized group challenged with H9N2 at 28 days post vaccination.

**Table 3:** Serum HA antibody levels in chickens vaccinated with the recombinant viruses.

Log2 HI Titer (Mean ± SD)	Days Post Vaccination (dpv)			
	7	14	21	28
HVT-005/006-MLV-HA	0	0.6 ± 0.55	2.8 ± 0.84	4.8 ± 0.84
HVT-005/006-hCMV-HA	0	0.8 ± 0.45	2.4 ± 0.55	4.4 ± 0.55
HVT-029/031-MLV-HA	0	0.6 ± 0.55	2.0 ± 0.71	4.2 ± 1.1
HVT	0	0	0	0

## Data Availability

Not applicable.
